# Morphology and molecules support the new monotypic genus *Fenghwaia* (Rhamnaceae) from south China

**DOI:** 10.3897/phytokeys.171.57277

**Published:** 2021-01-06

**Authors:** Gang-Tao Wang, Jiang-Ping Shu, Guo-Bin Jiang, Yu-Qiang Chen, Rui-Jiang Wang

**Affiliations:** 1 Key Laboratory of Plant Resources Conservation and Sustainable Utilization, South China Botanical Garden, Chinese Academy of Sciences, Guangzhou, Guangdong 510650, China South China Botanical Garden, Chinese Academy of Sciences Guangzhou China; 2 University of Chinese Academy of Sciences, Beijing 100049, China University of Chinese Academy of Sciences Beijing China; 3 Taicheng Town, Taishan, Jiangmen City, Guangdong 529200, China Unaffiliated Taishan China

**Keywords:** *
Fenghwaia
*, IUCN, palynology, Rhamnoid, taxonomy

## Abstract

*Fenghwaia*, a new monotypic genus, along with the new species *Fenghwaia gardeniicarpa*, is described from Guangdong Province, China. The combined features of inferior ovary, cylindrical drupaceous fruits and orbicular and dorsiventrally-compressed seeds with an elongate and pronounced basal appendage make the new genus significantly different from other genera of the family. In addition, its pollen morphology also showed great similarity to other species of this stenopalynous family. The molecular phylogenetic analysis, based on nuclear ribosomal internal transcribed spacer (ITS) and plastid *trnL-F* intron spacer (*trnL-F*) DNA sequence data from the new genus and the other 375 species representing 58 genera of Rhamnaceae, indicates that *Fenghwaia* is nested within the ‘rhamnoid’ group and sister to the tribe Rhamneae and then both sister to the tribe Maesopsideae. A taxonomic classification key to the ‘rhamnoid’ group is provided, based on morphological characters. A global conservation assessment is also performed and classifies *Fenghwaia gardeniicarpa* as Near Threatened (NT).

## Introduction

The buckthorns (Rhamnaceae Juss.) are a cosmopolitan family of small trees, shrubs, climbers and, occasionally, herbs and are well represented both in tropical and temperate regions (Raven and Axelrod 1974). This family includes approximately 900 species belonging to about 60 genera and 11 tribes. China hosts 13 genera and 137 species, distributed throughout the whole country, but mostly in south and southwest China (Chen and Schirarend 2007). The species of the family are mainly characterised by having basically cymose inflorescence mostly in the axillary position, usually 4–5-merous flowers, superior to inferior 2–4-loculed ovary with one ovule in each locule and indehiscent schizocarpic or capsular fruits (Medan and Schirarend 2004).

Rhamnaceae was recently revealed to be a monophyletic family and a member of the order Rosales with other eight families, viz. Rosaceae, Barbeyaceae, Dirachmaceae, Elaeagnaceae, Ulmaceae, Cannabaceae, Moraceae and Urticaceae on the basis of molecular evidence (APG IV 2016). The systematic treatment of Rhamnaceae, based on molecular data, morphological, anatomical and geographical information, indicated that Rhamnaceae should divided into three groups, viz. the ‘rhamnoid’ group, ‘ziziphoid’ group and the ‘ampelozizyphoid’ group (Richardson et al. 2000a, 2004; Hauenschild et al. 2016b).

During our field investigation in 2018, a treelet of Rhamnaceae, morphologically similar to *Sageretia* species, but bearing cylindrical and more or less fleshy drupaceous fruits with an inferior ovary and persistent calyxes, somewhat similar to the capsules of *Gardenia*, was found along the riverside of a secondary broad-leaf forest adjacent to a reservoir in Taishan, Jiangmen City, Guangdong Province. This plant is very different from any known species and evoked great interest for clarifying the taxonomic name and its phylogenetic relationship. Several field expeditions from March to August 2019 were subsequently undertaken to clarify its inflorescence and flower characters. Phylogenetic analysis, based on ITS and *trnL-F*, along with morphological comparisons, suggested that this species is best treated as a new taxon belonging to a new genus of Rhamnaceae.

## Material and methods

All morphological data of the new species were collected by Light Microscope and Stereomicroscope. Palynological observations followed Guo and Wang (2011). The voucher specimens have been deposited at South China Botanical Garden, Chinese Academy of Sciences (IBSC).

Since nuclear ribosomal internal transcribed spacer region (ITS) and plastid *trnL-F* intron spacer region (*trnL-F*) were already shown to include sufficient information to reconstruct well-supported topologies in Rhamnaceae (Hauenschild et al. 2016a, b), hence a total of five genomic DNAs were extracted from five different individuals, using a modified cetyltrimethylammonium bromide (CTAB) method (Allen et al. 2006).

The primers and PCR protocols were outlined by Hauenschild et al. (2016a, b). The PCR products were sent to Sangon Biotech (Shanghai, China) and sequencing was conducted using an ABI 3730xl DNA Analyzer (Applied Biosystems, Invitrogen, Foster City, CA, USA).

Multiple locus alignment of 590 operational taxonomic units (OTUs) was performed by Mafft v 7.453 (Katoh et al. 2002) with default parameters and ambiguous positions in the alignment were removed by GBlocks v 0.91b with the parameters (-b4 = 5, -b5 = h) (Castresana 2000). After filtering, the best-fit model (TIM+F+R4) was selected on the basis of the Bayesian Information Criterion (BIC) using Modelfinder (Kalyaanamoorthy et al. 2017) and the phylogenetic tree with the Maximum Likelihood (ML) method was performed by IQ-TREE v1.6.12 (Nguyen et al. 2015). Ultrafast bootstrap values were calculated with 1000 random replicates (Hoang et al. 2018). For Bayesian Inference (BI), we used the GTR+G+I model and performed four independent Markov Chain Monte Carlo (MCMC) reactions in MrBayes v3.2.6 (Ronquist and Huelsenbeck 2003), running five million generations every Markov Chain, sampling one tree every 1,000 generations, rejecting 25% of the trees as burn-in after the value of average standard deviation of split frequencies was lower than 0.01.

## Results

All DNA data of 585 OTUs, representing 375 species of 58 genera and 11 Rhamnaceae tribes, released by Hauenschild et al. (2016a), were downloaded from GenBank and merged into our present analysis. The GenBank numbers of the newly-sequenced *Fenghwaia gardeniicarpa* are: *Y.Q.Chen & G.T. Wang* 1223-1, ITS: MN795061, *trnL-F*: MN793985; *Y.Q. Chen & G.T. Wang* 1223-2, ITS: MN795062, *trnL-F*: MN793986; *Y.Q. Chen & G.T. Wang* 1224-1, ITS: MN795063, *trnL-F*: MN793987; *Y.Q. Chen & G.T. Wang* 1224-2, ITS: MN795064, *trnL-F*: MN793988; *Y.Q. Chen & G.T. Wang* 1225, ITS: MN795065, *trnL-F*: MN793989.

**Figure 1.**
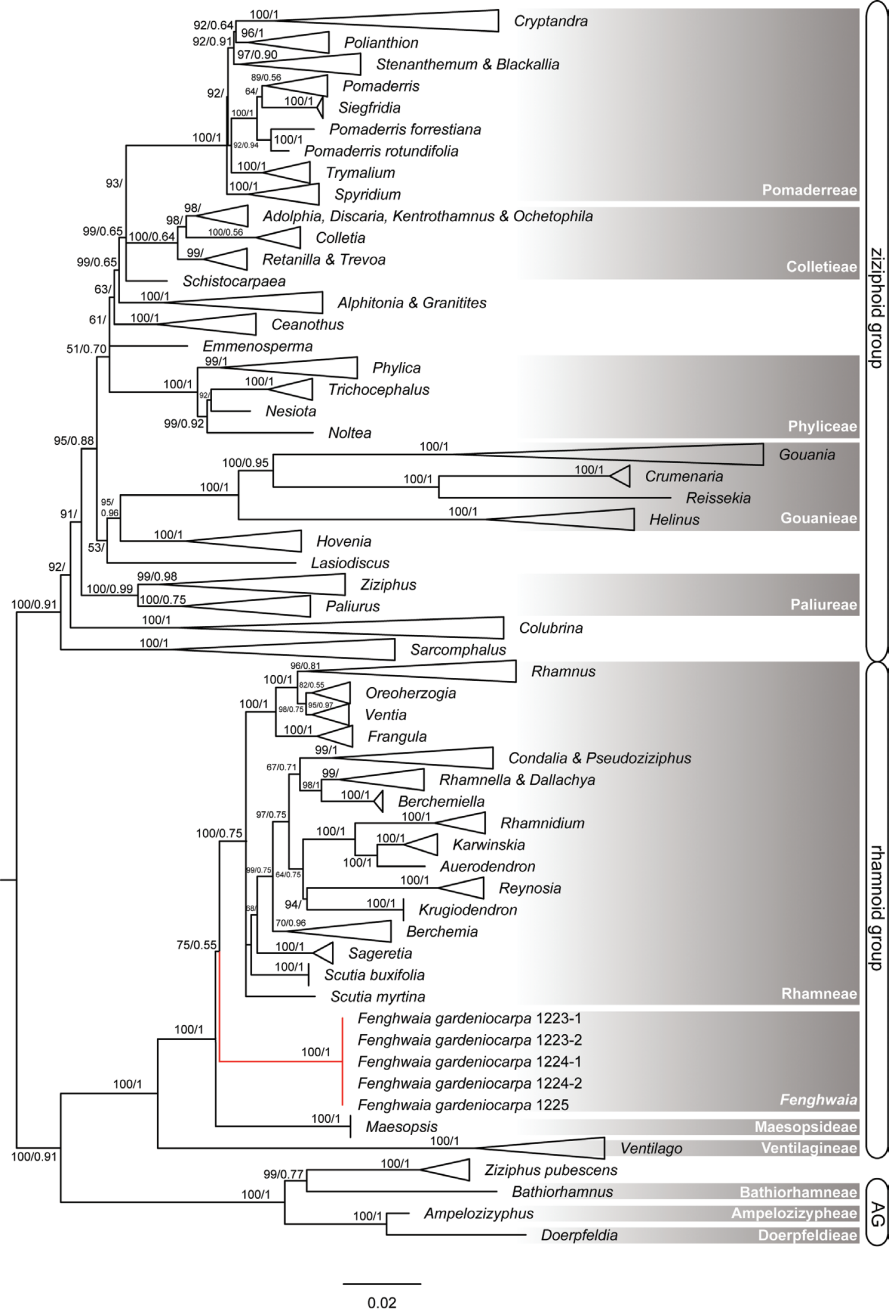
The phylogenetic consensus tree of Rhamnaceae with ML and BI methods, on the basis of ITS and *trnL-F* sequences. AG: ‘ampelozizyphoid’ group. The numbers above the branches are Maximum Likelihood support values (left) and MrBayes posterior probability (right).

The topology of our phylogenetic tree is similar to that of Hauenschild et al. (2016b), but the tribe Paliureae, defined by Hauenschild et al. (2016b) with very weak support, collapsed in our analysis, because of the exclusion of *Hovenia* and *Sarcomphalus*. The new genus *Fenghwaia* is nested into the ‘rhamnoid’ group and sister to the tribe Rhamneae with weak bootstrap support (BS/PP = 75/0.55), but then both sister to the tribe Maesopsideae with strong support (BS/PP = 100/1) (Fig. 1).

### Taxonomic description

#### Fenghwaia

Taxon classificationPlantaeRosalesRhamnaceae

G.T. Wang & R.J. Wang gen. nov.

A9BE3254-9A4C-5E71-BF54-2948B76AFC88

urn:lsid:ipni.org:names:77213613-1

##### Type species.

Fenghwaia gardeniicarpa G.T. Wang & R.J. Wang.

##### Diagnosis.

*Fenghwaia* is distinctly different from other genera by a character combination of its cucullate flowering petals, inferior ovary with 3-locular and one ovule in each locule, elongate capsular fruit with five longitudinal ridges and verrucose seeds.

A single species is only known from China.

##### Etymology.

We dedicate this new genus to Professor Chen Fenghwai, a Chinese plant taxonomist, in honour of his great contribution to the botanical gardens in China.

#### Fenghwaia gardeniicarpa

Taxon classificationPlantaeRosalesRhamnaceae

G.T. Wang & R.J. Wang sp. nov.

5AB389D2-F88F-5CF1-B5EA-41581DDAAB12

urn:lsid:ipni.org:names:77213614-1

Fig. 2

##### Type.

China. Guangdong Province, Jiangmen City, Taishan, Mt. Nanfengshan, under secondary mixed forests, 22°11'N, 112°56'E, elev. ca. 410 m, 6 July 2019, *R.J. Wang, G.T. Wang & G.B. Jiang 1228* (***holotype***: IBSC0849961; ***isotypes***: CSH0171170; IBK00421260; IBSC0849962; IBSC0849963; KUN1347949; PE02251842).

**Figure 2.**
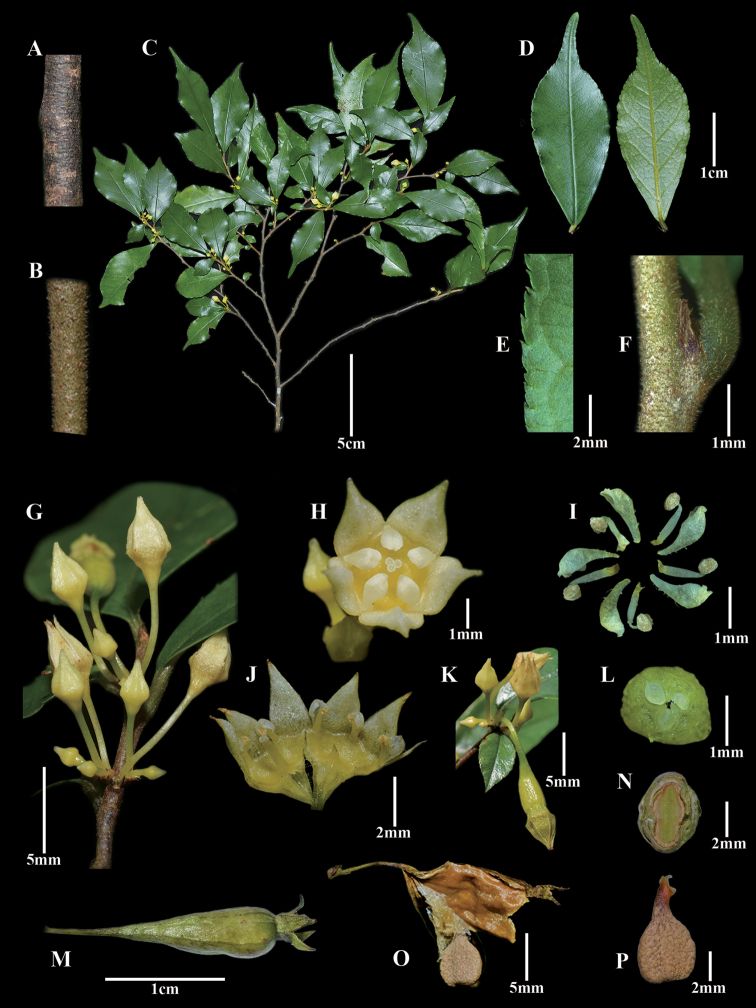
*Fenghwaia gardeniicarpa*: **A** main stem with glabrous surface **B** young stem with pubescent surface **C** fertile branches **D** adaxial (left) and abaxial (right) side of leaf blade, respectively **E** serrated leaf margin **F** stipule **G** inflorescence **H** flower in anthesis **I** morphology of petals and stamens **J** longitudinal section of a flower, showing the stamens enclosed by cucullate petals **K** young fruit **L** transection section of an ovary, showing three ovules **M** mature fruit **N** transection section of mature fruit with only one well-developed seed **O** dehiscent capsule **P** seed, with an elongate and pronounced basal appendage. Photos: G.T. Wang, G.B. Jiang.

##### Description.

Treelet, evergreen, 0.5–2 m tall, slender, much branched at top; main stems dark or brown, slender, glabrous; young branches rusty strigose at surface. Leaves alternate, anisophyllous, often clustering at the top of branches; petiole 2–5 mm long, pubescent; leaf blade 5.5–10.1 × 1.9–4.0 cm, elliptic, oblanceolate-elliptic or ovate, thinly leathery, glabrous both sides, acuminate to caudate at apex, cuneate at base; secondary veins 3–5 each side, mid-rib and secondary veins smooth adaxially and prominent abaxially; margin entire at base and then serrate to apex. Inflorescence in sessile or shortly peduncled, axillary cymes or small thyrses, 3–5-flowered; bracts ca. 0.5–1.0 mm long, lanceolate to broadly triangular, yellow to rusty. Flowers bisexual, actinomorphic, yellowish-green, ca. 4–5 mm in diameter; pedicels very short to 4–6 mm long, glabrous; calyx lobes five, 2.0–3.0 × ca. 1.5 mm, ovate triangular, depressed longitudinally at middle; hypanthium 2–3 mm long, slightly campanulate; petals five, ca. 1.5 mm long, cucullate, each partly covering the pollen-presenting surface of the anthers, shortly clawed at base, concaved at apex; stamens five, antepetalous, ca. 1.3 mm long, enclosed by petals; anthers ca. 0.3 mm long, ovoid, 4-locular, dorsifixed, filaments ca. 1.0 mm long; disc inconspicuous, adnate to the lower part of hypanthium; styles ca. 1.5 mm long, stigma 3-lobed; ovary inferior, 3-1ocular, with one ovule in each locule, ovules anatropous, basal, erect. Fruit drupaceous, more or less fleshy, cylindrical, ca. 1.5 cm long, 0.4–0.6 cm in diam., with five longitudinal ridges on surface, slowly dehiscent at top and then septicidally, glabrous; calyx lobes persistent. Seeds ca. 4 × 3 mm, orbicular, dorsiventrally compressed, brown, verrucose at surface, with an elongate and pronounced basal appendage.

##### Phenology.

Flowering from June to October; fruiting from August to December.

##### Palynology.

**Figure 3.**
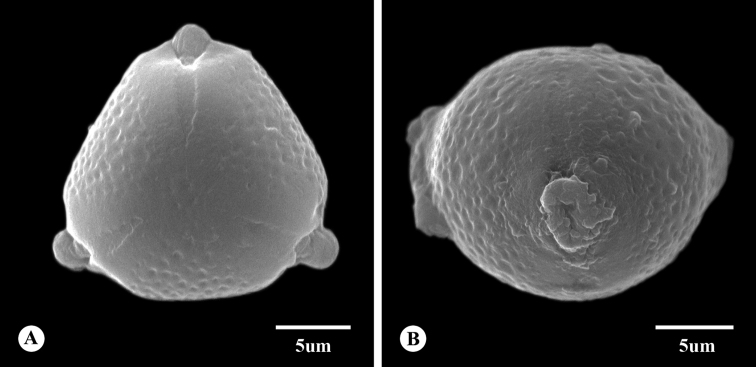
Pollen grains of *Fenghwaia gardeniicarpa*: **A** polar view **B** equatorial view.

The pollen grains of the new species are monads, isopolar, suboblate, radially symmetrical, angulaperturate, 3-zono-colporate apertures and psilate to perforate tectum. The pollen size is 14.9 (13.3–17.1) × 21.5 (16.1–21.1) μm and P/E value is 0.86 (Fig. 3).

##### Distribution and habitat.

*Fenghwaia gardeniicarpa* is endemic to mountains in Jiangmen District, Guangdong Province, China. It grows under secondary mixed forests at altitudes of 230–450 m, mountain slopes with 60–70% canopy density, accompanying herbal *Gahnia tristis* Nees (Cyperaceae) and *Adiantum flabellulatum* L. (Adiantaceae) and woody *Barthea barthei* (Hance ex Benth.) Krasser (Melastomataceae), *Dunnia sinensis* Tutcher (Rubiaceae) and *Illicium dunnianum* Tutcher (Schisandraceae).

##### Preliminary conservation assessment.

About 2000 mature *Fenghwaia gardeniicarpa* individuals from four localities have been found in less than 800 km^2^ up to now. This area can be classified as the extent of occurrence. The plants have no any ornamental or medicinal uses. They are well protected in a Nature Reserve and the population is not severely fragmented. In addition, no population decline and no extreme fluctuations caused by natural events have been observed in their habitats. According to the International Union for Conservation of Nature (2012) and IUCN Standards and Petitions Committee (2019), a category of Near Threatened (NT) is recommended for *Fenghwaia gardeniicarpa* for the present.

##### Etymology.

The species name highlights the striking resemblance with fruits of *Gardenia jasminoides* Ellis (Rubiaceae), an unusual and new feature for a fruit of Rhamnaceae.

##### Vernacular name.

Feng Huai Mu (Chinese pronunciation); 封怀木 (Chinese name).

##### Paratypes.

China: Guangdong Province, Jiangmen City, Taishan, Chixi Town, Tonggu Village, 21°55'N, 112°56'E, elev. 440 m, 2 June 2019, *Y.Q. Chen & G.T. Wang 1223, 1224, 1225, 1226* (IBSC!); Guangdong Province, Jiangmen City, Taishan, Chixi Town, Luobo Village, 21°55'N, 112°55'E, elev. 107 m, 20 June 2020, *R.J. Wang & Y.Y. Liu 5928* (IBSC!); Guangdong Province, Jiangmen City, Xinhui, Gudoushan Nature Reserve, 22°9'N, 112°55'E, elev. 231 m, 3 October 2019, *H.G. Ye et al. GDS-00849* (IBSC!).

## Discussion

### Palynology of *Fenghwaia gardeniicarpa*

Rhamnaceae is a stenopalynous family (Erdtman 1952). The pollen morphology of the Rhamnaceae usually has suboblate to oblate spheroidal or subprolate, a distinct triangular shape in polar view, oblate shape in equatorial view and 3-zonocolporate features. It does not show any special features and the descriptions usually concur with each other (Punt et al. 2003). The tectum of Rhamnaceae can be microreticulate, striate or rugulate to reticulate, baculate, verrucate, psilate, with more or less densely-spaced perforations and this variation of exine ornamentation has usually been used for classifying the pollen type (Schirarend and Köhler 1993; Medan and Schirarend 2004; Perveen and Qaiser 2005). In general, the main features of pollen grains of *Fenghwaia gardeniicarpa* are consistent with those of most other Rhamnaceae species with respect to the shape, polarity, symmetry, aperture number and position, size and tectum ornamentation (Medan and Schirarend 2004). However, the exine ornamentation of *Fenghwaia gardeniicarpa* is more similar to that of the tribe Rhamneae, as observed in *Berchemia* (reticulate), *Frangula*, *Rhamnus* (suprareticulate-rugulate, psilate), *Sageratia* (fossulate-perforate) than to that of the *Maesopsis*-type (baculate) (Punt et al. 2003; Perveen and Qaiser 2005; Naimat et al. 2012).

### Phylogenetic relationship of *Fenghwaia*

Based on a phylogenetic analysis of *rbcL* and *trnL-F* sequences of the plastid genome, Richardson et al. (2000b) outlined a new tribal classification of Rhamnaceae, recognising 11 tribes encompassing the informally-named groups of ‘rhamnoid’, ‘ampeloziziphoid’ and ‘ziziphoid’. Our phylogenetic analysis showed that *Fenghwaia* is nested within the ‘rhamnoid’ group. Thus, the ‘rhamnoid’ group consists of the tribes of Rhamneae, Maesopsideae and Ventilagineae and the genus *Fenghwaia*. The weak support (ML = 75/BI = 0.55) of the clade Rhamneae/*Fenghwaia* was probably caused by lack of adequate informative sites in the applied fragments. For example, only *trnL-F* sequences of *Maesopsis eminii* were applied in the present analysis. Morphologically, Rhamneae can be easily recognised by its fleshy fruit and 2- or 4-locular ovary. Maesopsideae and Ventilagineae have superior or half inferior ovaries, 1–2-locular, drupe (tribe Maesopsideae), samara or rostrate capsules (tribe Ventilagineae). Moreover, *Maesopsis* differs from all other genera in Rhamnaceae in its single-celled ovary and a style laterally attached to the fruit, rather than apically. Ventilagineae is unique in its fruits with a pronounced apical appendage (Richardson et al. 2000b).

*Fenghwaia* has an inferior and 3-loculed ovary, orbicular and dorsiventrally compressed seeds with an elongate and pronounced basal appendage, but its morphological characters are obviously different from those of other taxa in the ‘rhamnoid’ group. In addition, the genera that have an inferior ovary and 3-locular in Rhamnaceae are all in the ziziphoid group, viz. *Phylica*, *Trichocephalus* and *Nesiota* of the tribe Phyliceae, all genera of the tribe Gouanieae, *Siegfriedia*, *Spyridium* and *Cryptandra* of the tribe Pomaderreae, and *Alphitonia* and *Granitites* of the undefined tribe.

### A taxonomic classification key of the ‘rhamnoid’ group

**Table:** 

1	Ovary inferior, 3-locular; fruits dry, cylindrical, with ridges on the surface	*** Fenghwaia ***
–	Ovary superior, half-inferior or rarely inferior 1, 2-or 4-locular; fruits with wings, dry membranous rings or fleshy	**2**
2	Fruit an apically-winged samara or a rostrate capsule	** Ventilagineae **
–	Fruit a 1–4-locular drupe, not samara or a rostrate capsule	**3**
3	Ovary superior or half-inferior, 2- or 4-locular	**Rhamneae**
–	Ovary superior, 1-locular	** Maesopsideae **

## Supplementary Material

XML Treatment for Fenghwaia

XML Treatment for Fenghwaia gardeniicarpa
